# Characterisation of ascocorynin biosynthesis in the purple jellydisc fungus *Ascocoryne sarcoides*

**DOI:** 10.1186/s40694-022-00138-7

**Published:** 2022-04-27

**Authors:** Carsten Wieder, Roberta Peres da Silva, Jessica Witts, Christof Martin Jäger, Elena Geib, Matthias Brock

**Affiliations:** 1 Fungal Biology Group, School of Life Sciences, University of Nottingham, University Park, Nottingham, NG7 2RD UK; 2 Present Address: Institute of Molecular Physiology, Johannes-Gutenberg University Mainz, Hanns-Dieter-Hüsch-Weg 17, 55128 Mainz, Germany; 3 Present Address: University of Exeter, Stocker Road, Exeter, EX4 4QD UK; 4 Sustainable Process Technologies Research Group, Faculty of Engineering, University of Nottingham, University Park, Nottingham, NG7 2RD UK

**Keywords:** NRPS-like enzymes, Monooxygenase, Biosynthesis gene cluster, Heterologous gene expression, In vitro assays

## Abstract

**Background:**

Non-ribosomal peptide synthetase-like (NRPS-like) enzymes are highly enriched in fungal genomes and can be discriminated into reducing and non-reducing enzymes. Non-reducing NRPS-like enzymes possess a *C*-terminal thioesterase domain that catalyses the condensation of two identical aromatic α-keto acids under the formation of enzyme-specific substrate-interconnecting core structures such as terphenylquinones, furanones, butyrolactones or dioxolanones. *Ascocoryne sarcoides* produces large quantities of ascocorynin, which structurally resembles a terphenylquinone produced from the condensation of *p*-hydroxyphenylpyruvate and phenylpyruvate. Since the parallel use of two different substrates by a non-reducing NRPS-like enzyme appeared as highly unusual, we investigated the biosynthesis of ascocorynin in *A. sarcoides*.

**Results:**

Here, we searched the genome of *A. sarcoides* for genes coding for non-reducing NRPS-like enzymes. A single candidate gene was identified that was termed *acyN*. Heterologous gene expression confirmed that AcyN is involved in ascocorynin production but only produces the non-hydroxylated precursor polyporic acid. Although *acyN* is embedded in an ascocorynin biosynthesis gene cluster, a gene encoding a monooxygenase required for the hydroxylation of polyporic acid was not present. Expression analyses of all monooxygenase-encoding genes from *A. sarcoides* identified a single candidate that showed the same expression pattern as *acyN*. Accordingly, heterologous co-expression of *acyN* and the monooxygenase gene resulted in the production of ascocorynin. Structural modelling of the monooxygenase suggests that the hydrophobic substrate polyporic acid enters the monooxygenase from a membrane facing entry site and is converted into the more hydrophilic product ascocorynin, which prevents its re-entry for a second round of hydroxylation.

**Conclusion:**

This study characterises the first naturally occurring polyporic acid synthetase from an ascomycete. It confirms the high substrate and product specificity of this non-reducing NRPS-like enzyme and highlights the requirement of a monooxygenase to produce the terphenylquinone ascocorynin.

**Supplementary Information:**

The online version contains supplementary material available at 10.1186/s40694-022-00138-7.

## Introduction

**Fig. 1:**
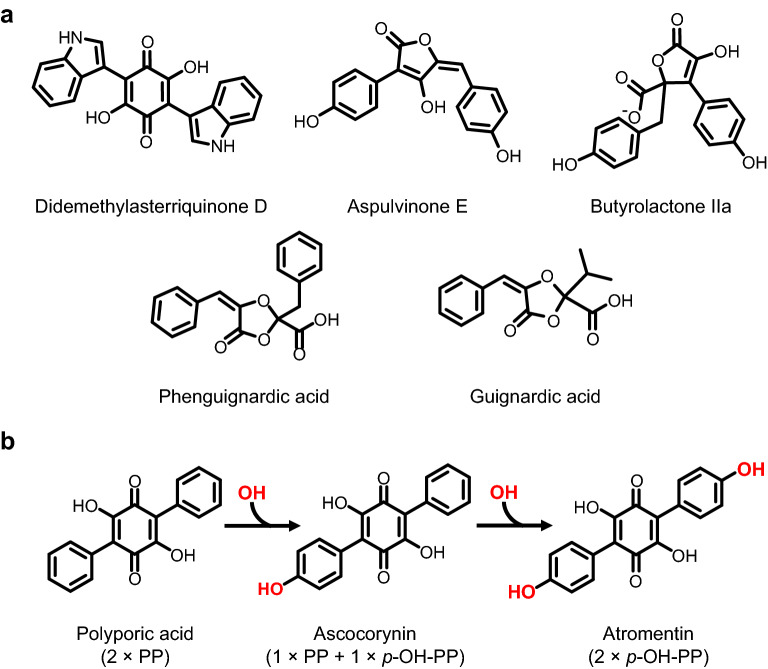
Selection of metabolites produced by NRPS-like enzymes. **a** The terpenylquinone didemethyltasterriquinone D, the furanone aspulvinone E, the butyrolactone butyrolactone IIa and the dioxolanone phenguignardic acid with its related compound guignardic acid. **b** Monohydroxylation of the terphenylquinone polyporic acid leads to ascocorynin and a second hydroxylation to atromentin. Alternatively, while polyporic acid is produced from two molecules of phenylpyruvate (PP), ascocorynin can be synthesised from one molecule of phenylpyruvate and one molecule of *p*-hydroxyphenylpyruvate (*p*-OH-PP), and atromentin can be produced from the condensation of two molecules of *p*-hydroxyphenylpyruvate

Genes coding for non-reducing non-ribosomal peptide synthetase-like (NRPS-like) enzymes are widely distributed among fungi. The metabolites produced by these enzymes contribute to a range of different biological activities such as phytotoxicity [1], quorum sensing [2] or the formation of protective pigments [3] to mention only a few. The domain structure of non-reducing NRPS-like enzymes consists of an adenylation (A), thiolation (T) and thioesterase (TE) domain [4] but they lack the condensation (C) domain that is vital for peptide bond formation in true non-ribosomal peptide synthetases. Thus, non-reducing NRPS-like enzymes are unable to produce peptide bonds and do not seem to use amino acids as substrates. Instead, in all NRPS-like enzymes characterised so far the A domain activates two identical aromatic alpha-keto acids that derive from either phenylalanine, tyrosine or tryptophan [4]. The T domain accepts the activated alpha-keto acids on its phosphopantetheine-loaded acyl carrier protein (ACP) and transfers the substrates to the TE domain that eventually performs a condensation reaction of the two substrate molecules [5]. While the exact reaction mechanism occurring in the active site of TE domains has not been elucidated in detail, condensation reactions result in a TE domain specific formation of substrate interconnecting core structures such as benzoquinones, furanones, butyrolactones or dioxolanones [4–6] (Fig. 1a). While the A domain is generally very specific for activating a single type of substrate [4, 6, 7], it has been speculated that there might be exceptions from this rule. The phytopathogenic fungus *Guignardia bidwellii* produces the dioxolanone phenguignardic acid [1] (Fig. 1a), which seems to derive from an NRPS-like enzyme as shown by studies on PngA (also named PgnA) from *Aspergillus terreus* [8]. However, in *G. bidwellii* the related dioxolanone guignardic acid (Fig. 1a) and other dioxolanone molecules have been identified which implies that besides phenylpyruvate the deamination products from valine, tyrosine and alanine might also be used as substrates [9]. Therefore, an NRPS-like enzyme in *G. bidwellii* is either capable of activating and condensing a variety of different substrates or the initial product phenguignardic acid becomes modified by a set of tailoring enzymes. Another example that implies the simultaneous use of mixed substrates derives from *Ascocoryne sarcoides*. *A. sarcoides* is an endophytic ascomycete that has gained attention by its ability to produce potential biofuels as it releases eight-carbon volatile compounds from lignocellulose degradation [10]. Besides the production of these volatiles, *A. sarcoides* produces large quantities of a coloured compound named ascocorynin (Fig. 1b) that gives the fruiting body of this ascomycete a pink to purple colour and led to its common name as purple jellydisc fungus [11]. Ascocorynin contains a quinone core with one phenyl- and one hydroxyphenyl-side chain attached and, as such, resembles an intermediate between atromentin and polyporic acid (Fig. 1b). The competitive advantage for *A. sarcoides* from producing large quantities of ascocorynin remains speculative as biological activity analyses only revealed very weak antibiotic activity against gram-negative bacteria, moderate antibiotic activity against gram-positive bacteria and no growth inhibitory effect on a range of selected yeasts and filamentous fungi [11]. However, the related terphenylquinone atromentin (Fig. 1b) is used by the fungus *Paxillus involutus* to produce involutin that is involved in extracellular Fenton reactions occurring during the degradation of organic matter [7, 12]. By contrast, polyporic acid is produced by *Hapalopilus* species such as *H. rutilans* that is a cause of mushroom poisoning [13]. Thereby, polyporic acid causes antiproliferative effects by inhibiting the dihydroorotate dehydrogenase [14]. Thus, ascocorynin may aid in wood degradation as reactive oxygen scavenger, act as anti-feeding compound, or may have other yet unknown properties. However, besides the lack of knowledge on the precise biological function of ascocorynin, its biosynthesis remained highly speculative. As mentioned, the structure of ascocorynin could derive from the simultaneous use of phenyl- and *p*-hydroxyphenylpyruvate as substrates of an NRPS-like enzyme [11]. Alternatively (Fig. 1b), ascocorynin could be produced by the mono-hydroxylation of polyporic acid, which could theoretically be followed by a second hydroxylation of the resulting ascocorynin to yield atromentin [11]. To address the question of ascocorynin production in *A. sarcoides*, we extracted *A. sarcoides* cultures to identify the benzoquinones produced, performed genome mining in the search for NRPS-like enzymes and aimed in the heterologous expression of candidate genes to analyse their products.

## Results

### Identification of terphenylquinones from *Ascocoryne sarcoides* species

**Fig. 2:**
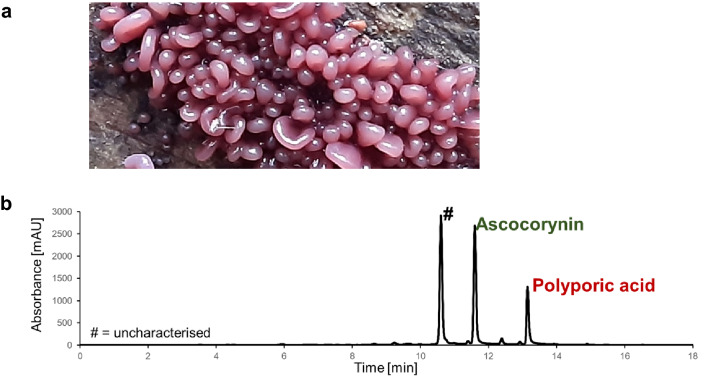
Metabolites produced by *A. sarcoides*. **a** Photograph of *A. sarcoides* growing on birch wood in Nottinghamshire (Photo kindly provided by P. Brett, University of Nottingham). Due to the purple colour of the fruiting bodies of *A. sarcoides* it is also known as the purple jellydisc fungus. **b** HPLC analysis of a culture extract from *A. sarcoides* CBS247.80 grown on potato dextrose agar. Ascocorynin and polyporic acid together with a not further characterised metabolite (#) are produced as main metabolites

To analyse the terphenylquinones produced by *A. sarcoides* species (Fig. 2a), strain NRRL 50072 and CBS 247.80 were grown for 5 days at 20 °C on potato dextrose agar plates, which resulted in a pink to purple colouration of the medium and the mycelium as expected from the production of terphenylquinones such as atromentin, ascocorynin or polyporic acid. Analysis of culture extracts by HPLC revealed three major metabolites (Fig. 2b). While one of the metabolites remains uncharacterised, the two other most prominent metabolites matched in retention time and UV/Vis profile to ascocorynin and polyporic acid, which was further confirmed by exact molecular mass determination with *m/z* 307.0616 [M−H]^−^ for ascocorynin and *m/z* 291.0664 [M−H]^−^ for polyporic acid. Atromentin was not detected in the culture extracts. These results indicated the presence of an NRPS-like enzyme that produces polyporic acid from phenylpyruvate but may also produce ascocorynin by the simultaneous use of *p*-hydroxyphenyl- and phenylpyruvate.

### Genome mining for NRPS-like enzymes

To identify NRPS-like enzymes capable of producing polyporic acid and ascocorynin, the genome of *A. sarcoides* strain NRRL 50072 [10] available at Mycocosm JGI was searched for NRPS-like enzymes in the secondary metabolism clusters annotation [15]. While the genome contains seven NRPS-like enzymes, only one of these enzymes with the gene and protein ID 6108 showed the expected A-T-TE domain structure of a non-reducing NRPS-like enzyme as confirmed by InterPro analysis [16]. To further verify that no other NRPS-like enzyme with the expected A-T-TE domain structure was present in the *A. sarcoides* genome, the protein sequence of transcript 6108 was used for a tBLASTn search [17] against the assembled genome nucleotide sequence. No other gene matching the required domain structure criteria was identified. Therefore, this enzyme served as prime candidate to produce terphenylquinones in *A. sarcoides* and we named this enzyme in subsequent experiments AcyN.

### Heterologous expression of *acyN* in *Aspergillus oryzae* OP12

**Fig. 3:**
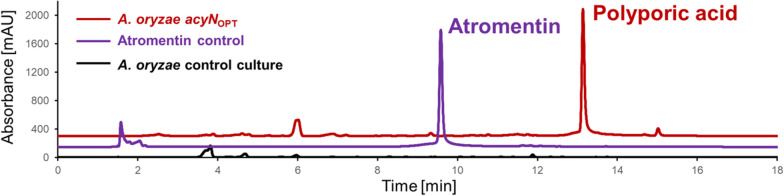
Metabolite analysis from *A. oryzae* OP12 expressing the codon-optimised *acyN*_OPT_ gene. No significant amounts of background metabolites are produced in the *A. oryzae* OP12 control strain. The strain expressing the *acyN*_OPT_ gene produces polyporic acid, but not ascocorynin or atromentin (the latter shown as reference). The same profile was observed for strains expressing the non-optimised *acyN* versions from strains NRRL50072 and CBS 247.80 (not shown)

To identify the product(s) formed by the non-reducing NRPS-like enzyme AcyN identified in the genome of *A. sarcoides*, we amplified the respective coding sequence from genomic DNA of *A. sarcoides* CBS 247.80 and from NRRL 50072. In addition, to avoid problems in heterologous protein production due to a codon bias, we generated a synthetic *acyN* gene (*acyN*_OPT_, GenBank Accession number OL770279) for codon-optimised heterologous protein production in the *Aspergillus oryzae* expression platform strain OP12_2Δ. *A. oryzae* OP12 contains a genomic copy of the transcriptional activator *terR* under control of the amylase promoter P*amyB*, which allows high-level expression of genes under control of the TerR target promoter P*terA* [6, 18]. Strain OP12_2Δ contains a partially deleted *pyrG* gene rendering the strain uridine/uracil auxotrophic and additionally contains a deletion of the *pabA* gene required for *p*-aminobenzoic acid biosynthesis. Using the *pyrG* marker in a first transformation allows a subsequent second transformation with the *pabA* marker without the need for a *pyrG* marker recycling. All different gene versions of the *acyN* gene were cloned into the *Nco*I site of plasmid SM-X_S-tag_URA, which is a derivative of plasmid *his*_SM-Xpress_URA [3, 6], but with a Strep-tag II sequence [19] replacing the His-tag sequence in *his*_SM-Xpress_URA. *A. oryzae* transformed with the *acyN* containing plasmids produced high levels of a purple compound on plates and in liquid culture regardless the source of the *acyN* gene indicating that all genes were functionally expressed. Culture extracts were analysed for terphenylquinone production and revealed the production of only polyporic acid, but not ascocorynin or atromentin (Fig. 3). This confirmed that AcyN is a functional NRPS-like enzyme but appears highly specific for the production of polyporic acid.

### Expression analysis of the ascocorynin biosynthesis gene cluster

**Fig. 4:**
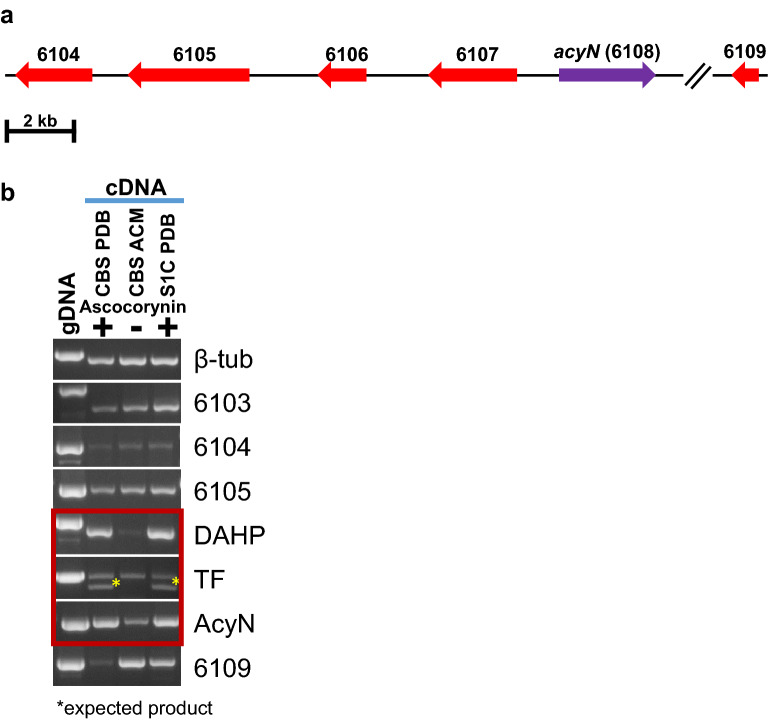
Expression analysis of genes surrounding the polyporic acid synthetase gene *acyN*. **a** Scheme of the genomic situation in strain NRRL 50072. Gene IDs: 6104 = Glycosyl hydrolase; 6105 = Phosphatidic acid-preferring phospholipase A1; 6016 = 3-Desoxyarabinoheptulosanate-7-phosphate (DAHP) synthase; 6107 = Fungal specific transcription factor (zinc ion binding); 6108 = NRPS-like enzyme with *C*-terminal thioesterase domain (AcyN); 6109 = Ribosomal protein L1 (intergenic region between 6108 and 6109 ~ 7 kb). **b** Semiquantitative RT-PCR analysis of genes 6103 to 6109 normalised against beta-tubulin gene expression levels. cDNA was generated from *A. sarcoides* strain (CBS 247.80) grown on either ascocorynin inducing potato dextrose broth (PDB) or non-inducing *Aspergillus* complete medium (ACM). Strain S1C was isolated from fruiting bodies of a strain found growing on birch wood in Nottinghamshire (UK, see also Fig. 2) and was cultivated on PDB medium only. Genomic DNA (gDNA) served as PCR control. The putative transcription factor (TF, gene ID 6107) shows basal unspliced constitutive expression. The spliced product (*) is only observed under inducing conditions. Only the genes coding for the DHAP synthase, the transcription factor and AcyN show an expression pattern that correlates with ascocorynin production

Since *acyN* expression in *A. oryzae* only resulted in the production of polyporic acid, we were interested in the expression of the genes surrounding *acyN* as they might form an ascocorynin biosynthesis gene cluster. An inspection of the genes adjacent to *acyN* (ID 6108) in the genome of strain NRRL 50072 (Fig. 4a) revealed no significant hit for a protein-coding region in the downstream region as confirmed by a manual blastx analysis [17] against the non-redundant protein database and the next gene named 6109 was found in a distance of about 7 kb and was annotated as ribosomal protein L1. By contrast, in the upstream region the adjacent gene 6107 was annotated as zinc-binding fungal specific transcription factor and gene 6106 was annotated as a putative 3-desoxyarabinoheptulosanate-7-phosphate (DAHP) synthase. These genes were followed by a phosphatidic acid-preferring phospholipase A1 (6105) and a glycosyl hydrolase (6104). While genes 6105 and 6104 may not belong to the biosynthesis gene cluster, the transcription factor 6107 could be responsible for *acyN* gene expression. Furthermore, DAHP synthases form the key initial step in the shikimate pathway of aromatic amino acid biosynthesis [20]. Therefore, gene 6106 could elevate the substrate levels for *acyN*. To analyse the expression of the genes in dependence of ascocorynin production, we observed that cultivation of *A. sarcoides* in PDB medium induced a rapid and strong purple colour formation, whereas, despite similar growth support, colour formation on *Aspergillus* complete medium was strongly delayed. Accordingly, RNA was isolated from strain CBS 247.80 grown under both conditions and from an *A. sarcoides* isolate collected in Nottinghamshire (strain S1C) also grown on PDB medium. RNA was reverse transcribed into cDNA and semiquantitative PCR was performed to analyse the expression pattern of all genes from ID 6103 to 6109 using the beta-tubulin gene as a control for normalisation of expression levels (Fig. 4b). Indeed, the DAHP synthase and *acyN* showed strong expression in PDB medium but not in ACM. Similarly, a band for the transcriptions factor at the expected cDNA size after excision of intron sequences was observed only under inducing conditions in PDB medium. However, it is worth to mention that an intron-containing transcript of the transcription factor was present under both, inducing and non-inducing conditions, which implies that the gene of the transcription factor is constitutively expressed, but intron splicing only occurs under inducing conditions. In conclusion, transcription analyses indicate that *acyN* combined with the transcription factor and the DAHP synthase gene are co-expressed and form a biosynthesis gene cluster. Other genes in the direct proximity show an expression pattern that is independent from pigment formation, which indicates that they do not belong to the *acyN* biosynthesis gene cluster.

### In vitro product formation from purified recombinant AcyN

**Fig. 5:**
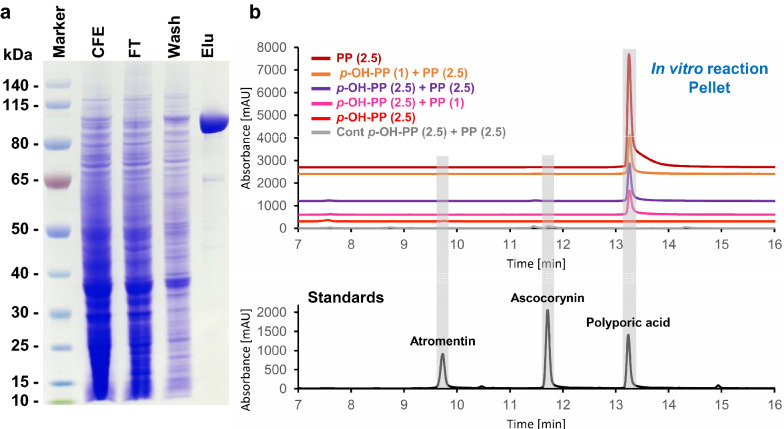
Purification of recombinant AcyN and in vitro characterisation of metabolite production. **a** SDS-PAGE analysis of Strep-tactin purified AcyN from *A. oryzae* OP12. CFE = cell free extract, FT = column flow through, Wash = column wash fraction, Elu = elution fraction. **b** HPLC analysis of extracts from the acid-precipitated pellet fractions from in vitro assays with purified AcyN. Numbers in brackets denote the concentration of substrates in [mM]. P, phenylpyruvate, p-OH-PP, *p*-hydroxphenylpyruvate, Cont, control reaction in the absence of AcyN. Positions of standards are indicated by grey bars. For the analysis of the supernatant extracts from in vitro reaction refer to Additional file 1: Fig. S1

Although only polyporic acid was identified as a product from recombinant expression of *acyN* in *A. oryzae* OP12, the putative DAHP synthase from the ascocorynin biosynthesis gene cluster could potentially elevate the intracellular levels of the two aromatic alpha-keto acids *p*-hydroxyphenyl- and phenylpyruvate to such an extent that it forces the enzyme to use both substrates in parallel. Therefore, we purified AcyN via the added Strep-tag II sequence (Fig. 5a) and subjected the purified enzyme to different substrate combinations under defined in vitro conditions. Acidification of the assays resulted in the precipitation of PIPES, protein and hydrophobic compounds, whereas the supernatant fraction mainly contains unreacted substrates and other more hydrophilic compounds (Fig. 5b and Additional file 1: Fig. S1). When the enzyme was incubated in the presence of *p*-hydroxyphenylpyruvate as the sole alpha-keto acid substrate, a trace amount of atromentin was detected in the supernatant fraction (Additional file 1: Fig. S1). By contrast, the use of phenylpyruvate as sole substrate resulted in the expected production of polyporic acid that was present in the pellet fraction (Fig. 5b). However, when both alpha-keto acids were mixed and regardless the concentration of the individual alpha-keto acids, only polyporic acid, but no ascocorynin or atromentin was detected (Fig. 5b and Additional file 1: Fig. S1). This confirmed the high specificity of AcyN as polyporic acid synthetase, but also implied that an additional enzyme that is not encoded in the ascocorynin biosynthesis gene cluster is involved in ascocorynin biosynthesis.

### Expression analysis of monooxygenase encoding genes in *A. sarcoides*

**Fig. 6:**
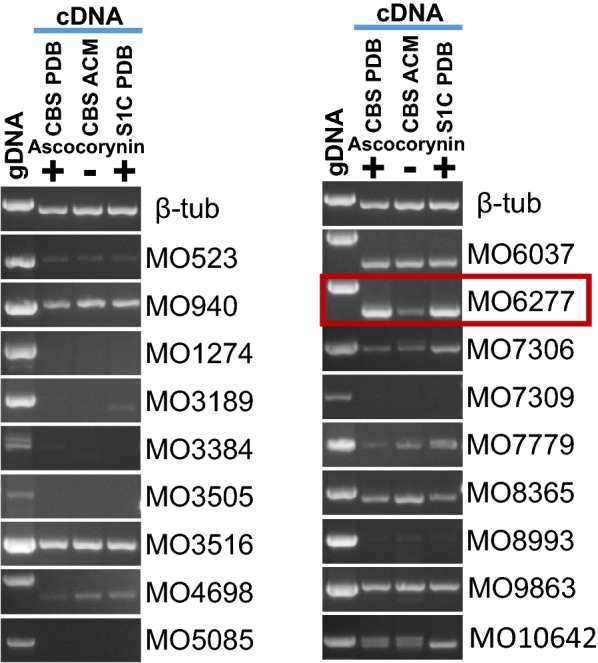
Semiquantitative RT-PCR analysis of all monooxygenase-encoding genes from *A. sarcoides*. The same cDNA from *A. sarcoides* strain (CBS 247.80) and strain SC1 was used as in the analysis of the ascocorynin biosynthesis gene cluster expression analysis. Potato dextrose broth (PDB) acts as ascocorynin-inducing and *Aspergillus* complete medium (ACM) as non-inducing condition. The beta-tubulin gene served for normalisation of cDNA concentrations and genomic DNA (gDNA) was used as PCR control. Only the monooxygenase MO6277 (boxed) shows the same expression pattern as the *acyN* gene

Our results confirmed that AcyN is highly specific to produce polyporic acid and the co-expressed genes upstream of *acyN* activate expression and could increase the levels of aromatic alpha-keto acids, but the combined action of the encoded proteins is insufficient to produce ascocorynin. Therefore, it appeared likely that an enzyme that is encoded outside the biosynthesis gene cluster performs the monohydroxylation of polyporic acid to yield ascocorynin. The most likely candidate to perform such a modification is a monooxygenase. However, within at least 50 genes upstream and downstream of *acyN* no monooxygenase was detected. Therefore, we screened the genome of *A. sarcoides* for all putative monooxygenase genes and eventually identified 18 candidate genes. To predict the most suitable candidate(s), we used the cDNA from the biosynthesis gene cluster analysis to screen for monooxygenase encoding genes with an expression pattern like that of the AcyN and DAHP synthase coding genes (Fig. 6). Ten of the monooxygenase encoding genes showed a constitutive expression pattern and seven genes showed no significant expression under the applied cultivation condition. However, the monooxygenase encoding gene with the ID 6277 revealed the same expression pattern as the genes from the ascocorynin biosynthesis gene cluster. Therefore, MO6277 was selected as prime candidate for the hydroxylation of polyporic acid to yield ascocorynin.

### Ascocorynin production from heterologous co-expression of *acyN* and MO6277

**Fig. 7:**
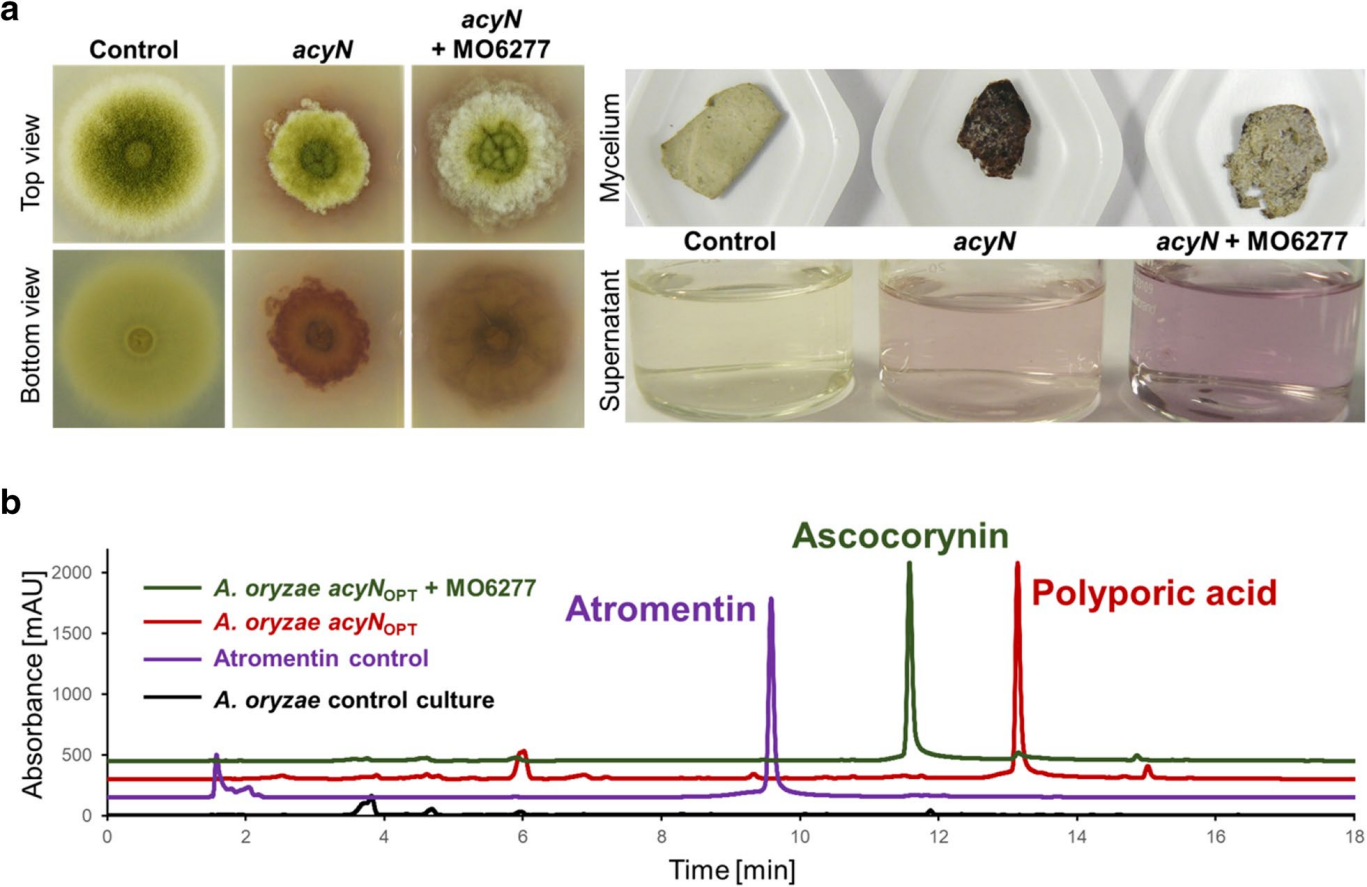
Phenotypic and metabolite analysis of *A. oryzae* OP12 strains expressing the polyporic acid synthetase gene *acyN* with or without co-expression of the MO6277 gene. **a** Strains were either grown for 60 h on potato dextrose agar plates (left) or for 24 h in liquid minimal medium with 2% starch as carbon source (right). An *A. oryzae* OP12 strain without expression construct served as control. When only the *acyN* gene is expressed, growth on plates and mycelium formation in liquid medium is strongly retarded and polyporic acid strongly attaches to the mycelium. Co-expression of the monooxygenase partially restores growth, and the produced pigments show increased solubility in the medium. **b** HPLC analysis of extracts from strains shown in (**a**). Polyporic acid produced in the *A. oryzae* strain expressing only the *acyN*_OPT_ gene is nearly quantitatively converted into ascocorynin. None of the strains produces atromentin (added as control)

To analyse the potential contribution of the monooxygenase MO6277 in ascocorynin production, we aimed in the co-expression of the gene in an *A. oryzae* OP12_2Δ strain expressing the codon-optimised synthetic *acyN* gene and produced high levels of polyporic acid. The MO6277 gene was cloned into an SM-X_S-tag_pabA plasmid to utilise the second auxotrophy of the expression platform strain. As expected, all transformants produced a purple colour, but the pigment appeared to have an increased solubility and showed less attachment to the mycelium (Fig. 7a). When the pigment was extracted and analysed by HPLC, ascocorynin was the major metabolite produced with only traces of polyporic acid remaining (Fig. 7b). These results confirm that MO6277 is a monooxygenase that hydroxylates polyporic acid and is responsible for the final step in ascocorynin production. Furthermore, as only ascocorynin but no atromentin was produced, it appears that the monooxygenase only accesses polyporic acid, but not ascocorynin as use of the latter substrate would result in the production of atromentin.

### In silico characterisation of MO6277

**Fig. 8:**
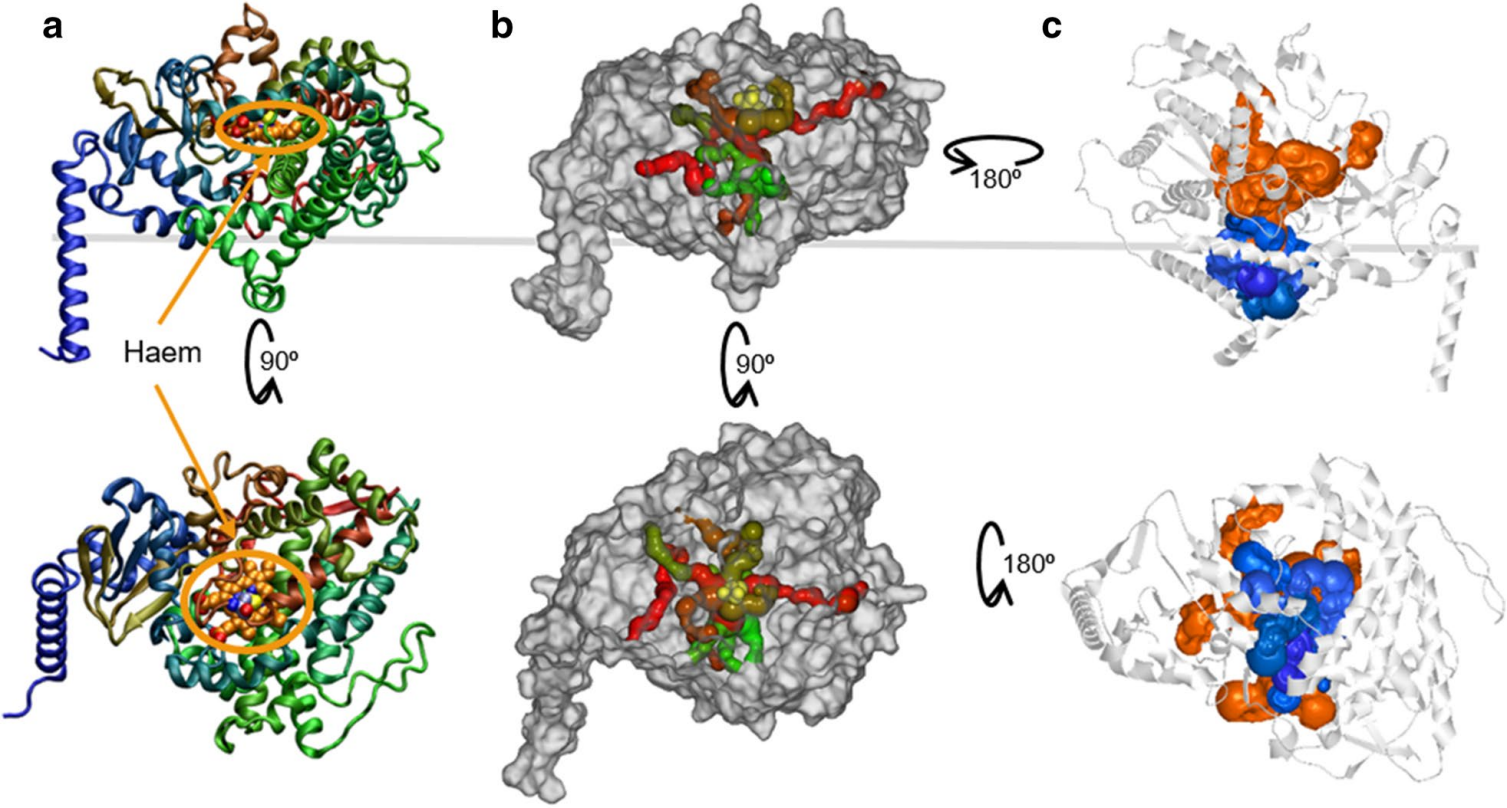
Structural model of the monooxygenase MO6277. **a** Alphafold structure model of MO6277 depicting a single membrane-spanning *N*-terminal alpha-helix and a buried haem binding site (docked haem model shown in orange van der Waals spheres). The potential location of the membrane surface is depicted as grey line. Secondary structure elements are coloured by sequence (BGR). **b** Substrate tunnels to the haem-containing binding site as calculated using CAVER software. Tunnels are coloured by priority as determined by the narrowest bottleneck from green (high priority) to red (low priority). **c** The most prominent tunnels to the active site from the membrane (blue) and cytosol facing side (orange)

Due to its essential contribution in ascocorynin production, we aimed in a more detailed computational analysis of the monooxygenase MO6277. We performed an initial BLAST search [17] against the non-redundant GenBank CDS. All returned hits were annotated as CYP450 monooxygenases and at least the top 100 protein sequences referred to enzymes of fungal origin. While this was not unexpected, the highest sequence identity was only 63% (BLAST similarity score of 714) and was to a cytochrome P450 monooxygenase from the ascomycete *Chalara longipes* BDJ (GenBank Accession number KAE9375486.1). This implies that MO6277 has specifically adapted to ascocorynin production, which has not been described from other fungal species. In a search against all characterised proteins in the protein data bank (pdb) the highest sequence identity of 26% was found against human microsomal CYP450 enzymes (e.g. 3A5, 3A7) that possess an *N*-terminal helix typical for membrane attached CYP450s [21]. Despite the low sequence identity to crystallised CYP450 enzymes, we generated a structural model using the DeepMind Alphafold2 software [22]. The modelled structure of MO6277 shown in Fig. 8 confirms the presence of an *N*-terminal alpha-helix and shows the typical deeply buried thiolate-bound haem-containing binding site in the predicted active site, in which a haem molecule has been manually modelled (Fig. 8a). This indicates that MO6277 belongs to the membrane-associated type of microsomal monooxygenases that can act on membrane associated substrates as observed for the monooxygenation of xenobiotics by human microsomal CYP450 enzymes [21]. This leads to the assumption that in MO6277 the hydrophobic substrate polyporic acid may enter the enzyme from the microsomal membrane. This assumption is further supported by investigation of possible substrate tunnels using the CAVER software on this static model structure [23]. While the structural fold of CYP450 enzymes is highly dynamic [21], the model shows better active site accessibility from the side of the enzyme that is most likely membrane attached (Fig. 8b, c). Therefore, a hydrophobic substrate such as polyporic acid might approach the active site from the membrane and, after hydroxylation, leaves into the cytoplasm as described for many other examples [21].

## Discussion

In this study we investigated the biosynthesis of the terphenylquinone ascocorynin in *A. sarcoides* to identify the responsible enzyme(s) and to answer the question on the substrates used for its biosynthesis. Besides ascocorynin, polyporic acid was identified from culture extracts of *A. sarcoides* (Fig. 2b). Since polyporic acid has previously been identified as product from the NRPS-like enzymes EchA in *Streptomyces* sp. LZ35 [24], it appeared likely that an active NRPS-like enzyme produces at least polyporic acid, but potentially also ascocorynin in *A. sarcoides*. The latter option was of specific interest as it had provided the first confirmed example of an NRPS-like enzyme that uses two different substrates in parallel for product formation. As only a single NRPS-like enzyme named AcyN was identified from the genome of *A. sarcoides* strain NRRL 50072, we used this gene and the surrounding gene loci for further investigation. As shown for other NRPS-like enzymes from ascomycetes such as TdiA, MicA, MelA or AbrA [3, 6, 25, 26], only a single product is formed from AcyN with a high specificity to produce polyporic acid. This formation of polyporic acid was independent of the source of *acyN* as our synthetic version of the gene, the original *acyN* gene from NRRL 50072 and the gene from CBS247.80 all produced significant levels of only polyporic acid in the heterologous expression host *A. oryzae*.

Purification of AcyN was easily achieved by exploiting the Strep-tag II sequence introduced at the *N*-terminus of the protein and chromatography over Strep-tactin columns resulted in highly pure enzyme preparations in a single purification step. Our in vitro studies on purified AcyN confirmed that confrontation of the enzyme with mixed substrates did not change the specificity for polyporic acid production. However, use of *p*-hydroxyphenylpyruvate resulted in the production of trace amounts of atromentin, but this was not observed in the presence of phenylpyruvate as substrate. While not quantified from several independent enzyme assays, it appears that the parallel presence of both substrates reduced product formation. This implies that *p*-hydroxyphenylpyruvate acts more like a competitive inhibitor rather than a substrate and the enzyme specifically produces polyporic acid. As the most likely route of ascocorynin production was via the action of a non-reducing NRPS-like enzyme, but AcyN appears to be the only NRPS-like enzyme with an A-T-TE domain structure in *A. sarcoides*, a tailoring enzyme is required to induce the hydroxylation of one of the two phenyl-side chains of polyporic acid.

Investigation of the expression of genes in proximity to *acyN* identified two co-expressed genes encoding a transcription factor and a gene coding for a putative desoxyarabinoheptulosanate-7-phosphate (DAHP) synthase, but no monooxygenase encoding gene, which was the most likely candidate for the monohydroxylation of polyporic acid. However, the identification of a co-expressed DAHP synthase is interesting per se as it mirrors the situation in the terrequinone A biosynthesis gene cluster from *A. nidulans* [25, 27]. Terrequinone A derives from modifications of didemethylasterrequinone D, which is the direct product from the non-reducing NRPS-like enzyme TdiA that uses indole pyruvate as substrate. The *tdiD* gene within the biosynthesis gene cluster encodes a l-tryptophan:phenylpyruvate aminotransferase, which allows the provision of elevated indole pyruvate substrate concentrations for TdiA by transamination of the amino acid l-tryptophan [25]. DAHP synthases catalyse the first reaction in the seven-steps of the Shikimate pathway required for the biosynthesis of the aromatic amino acids tyrosine, phenylalanine and, in a parallel pathway, tryptophan by transferring phosphoenolpyruvate to d-erythrose 4-phosphate [28]. The accompanied release of phosphate from the phosphoenolpyruvate substrate makes the reaction irreversible under physiologic conditions and directs the flux of carbon into aromatic amino acid biosynthesis. It is known that bacteria and fungi contain several isoforms of DAHP synthases that are mainly feedback regulated by aromatic amino acids [29, 30] to control the carbon flux into the Shikimate pathway. Given that polyporic acid is the precursor of ascocorynin and that the purple colour of fruiting bodies from *A. sarcoides* derives from large quantities of this compound, its biosynthesis requires a strong carbon flux towards the Shikimate pathway and is in complete agreement with a DAHP synthase co-regulated with *acyN* gene expression.

Due to the lack of a monooxygenase in proximity to the ascocorynin biosynthesis gene cluster, we performed expression analyses on monooxygenase encoding genes and identified the prime candidate MO6277 that showed the identical expression pattern as the genes of AcyN, the DAHP synthase and the transcription factor. While we cannot exclude that MO6277 acts on substrates other than polyporic acid, its essential contribution to ascocorynin biosynthesis was confirmed by co-expression with the *acyN* gene in *A. oryzae*, which resulted in the production of ascocorynin, but not the di-hydroxylated polyporic acid derivative atromentin. While it was unexpected that the gene was not located within the cluster of ascocorynin biosynthesis genes, a similar situation is found in the biosynthesis of NRPS-like enzyme-derived butyrolactones in *A. terreus*. BtyA is responsible for the production of the metabolite butyrolactone IIa [31]. Conversion into the quorum sensing active metabolite butyrolactone I [2] requires an O-methylation to butyrolactone II followed by a prenylation to yield butyrolactone I [32]. While a methyltransferase capable of methylating the carboxyl-group of butyrolactone IIa is found adjacent to the gene encoding the NRPS-like enzyme BtyA in *A. terreus* [32] and shares a bidirectional promoter, a prenyltransferase is not present in close proximity, but found adjacent to the aspulvinone H synthetase gene *apvA* in *A. terreus*. It has been experimentally confirmed that this transferase prenylates both, butyrolactone II and aspulvinone E [32]. Whether the monooxygenase MO6277 also hydroxylates substrates other than polyporic acid in *A. sarcoides* is currently unknown but it shows that tailoring enzymes acting on NRPS-like-derived metabolites do not necessarily need to be clustered.

While the lack of atromentin in our metabolite analysis from strains co-expressing *acyN* and *MO6277* does not exclude that ascocorynin can be used as a substrate under in vitro conditions to yield atrometin, its production appears non-favoured under in vivo conditions. By contrast, atromentin synthetases have been identified from various asomycetes and basidiomycetes and these enzymes directly and nearly exclusively use *p*-hydroxyphenylpyruvate as sole substrate to produce atromentin [6, 33, 34]. It should be mentioned that polyporic acid is highly hydrophobic and already starts to precipitate from methanol or acetonitrile during short-term storage. In addition, polyporic acid is strongly associated with the fungal mycelium as visualised by the dark coloured mycelium of *A. oryzae* strains expressing the *acyN* gene (Fig. 7a). Therefore, it is highly likely that polyporic acid produced in the cytoplasm attaches and solves in fungal membranes. Despite the use of the Alphafold2 software [22] that does not rely on homology models, the structure prediction of MO6277 may not represent the correct positioning of each structural element in detail. However, it clearly confirms the likely membrane association of the enzyme that is required for interaction with CYP450 reductases for electron transfer reactions [21]. In addition, the substrate tunnel prediction on this model using the CAVER software [23] indicates that the most likely entry side for substrates to approach the membrane facing catalytic centre of the haem cofactor is via the membrane facing site of the monooxygenase (Fig. 8b). This is also known for human microsomal monooxygenases that are involved in the detoxification of xenobiotics [21]. These monooxygenases hydroxylate a hydrophobic drug that is attached to or solved in the membranes. The hydroxylation produces a more polar compound that enters the second phase of a membrane-independent detoxification processes [35]. Similarly, compared to polyporic acid, ascocorynin is a much more hydrophilic compound and shows reduced attachment to the fungal mycelium as visualised by the reduced colouration of mycelium producing both, the polyporic acid synthetase and the monooxygenase. As co-expression of both genes in *A. oryzae* restored colony growth on solid media and mycelium formation in liquid medium (Fig. 7a), MO6277 may also be used for detoxification of polyporic acid in the natural ascocorynin producer *A. sarcoides*.

## Conclusions

In conclusion, while AcyN acts like other non-reducing NRPS-like enzymes by using two identical aromatic alpha-keto acids as substrates, the product polyporic acid is subsequently converted into the more polar and, thus, less toxic metabolite ascocorynin. While the monooxygenase encoding gene does not cluster with other enzymes involved in ascocorynin biosynthesis, it is co-regulated in *A. sarcoides* and appears vital to avoid high-level accumulation of polyporic acid in the fungal membrane.

## Methods

### Metabolite extraction from *A. sarcoides* cultures and metabolite analysis

Metabolite extractions from fungal culture supernatants and fungal mycelium were performed by acidification with 0.1% formic acid and ethyl acetate extraction followed by solvent evaporation under reduced pressure. Agar plates were extracted by cutting the agar into 1 cm^2^ large pieces that were transferred to glass bottles, overlayed with ethylacetate and sonicated in a sonication water bath for 30 min. Water was trapped by the addition of sodium sulfate and the ethylacetate phase was evaporated. The dried extracts were solved in methanol and subjected to HPLC analysis on a Dionex UltiMate3000 (Thermo Fisher Scientific) system using an analytical Eclipse XDB C18 column (Agilent, 250 mm × 4.6 mm, particle size 5 µm) as previously described [6]. High-resolution molecular mass analysis was performed in negative mode using a Bruker impact II Ultra-High-Resolution LC–QTOF MS with an Eclipse XDB C18 column as specified above.

### Kits and enzymes for molecular biology

All oligonucleotides and the synthetic gene used in this study were synthesised by Eurogentec (Kaneka Eurogentec S.A., Seraing, Belgium) and DNA sequences of all primers and their purpose are given in Additional file 2: Table S1. The proofreading Phusion DNA polymerase was used for gene cloning purposes, whereas Phire Hot start II polymerase was used in control PCR reactions (both Thermo Fisher Scientific, UK) with dNTPs from the Deoxynucleotide (dNTP) Solution Mix (New England Biolabs UK Ltd, Hitchin, UK). Restriction digests were performed by using FastDigest restriction enzymes and FastAP was used for dephosphorylation of plasmids when required (both Thermo Fisher Scientific, UK). DNA ligation reactions were performed by using the Rapid DNA ligation kit (Roche, Sigma-Aldrich, UK). In vitro recombination for plasmid assembly was performed using the InFusion HD cloning kit (Takara Bio Europe SAS, Saint-Germain-en-Laye, France). DH5α *Escherichia coli* cells were used to amplify plasmids and were made chemically competent using the Mix & Go *E. coli* Transformation Kit & Buffer Set and DNA fragments were isolated using the Zymoclean Gel DNA Recovery Kit (both from Zymo Research, Cambridge Bioscience, Cambridge, UK). Total RNA was purified using the MasterPure Yeast RNA Purification Kit (Epicentre, Lucigen, Cambio Ltd, Cambridge, UK) and additional DNase treatments were performed using the DNA-free DNA Removal Kit and first-strand cDNA synthesis was performed using Superscript III reverse transcriptase (both from Invitrogen, Thermo Fisher Scientific, UK).

### Generation of the *A. oryzae* OP12_2Δ strain

The *pyrG* negative *A. oryzae* expression platform strain OP12 *pyrG*^−^ [6] was used as parental strain for deletion of the *pabA* gene that is required for 4-amino-4-deoxychorismate synthesis in the *p*-aminobenzoic acid biosynthesis pathway. OP12 *pyrG*^−^ is a derivative *of A. oryzae* RIB40 and contains a genomic copy of the transcriptional activator *terR* from *A. terreus* under control of the *A. oryzae amyB* promoter [6]. In addition, its *pyrG* gene contains a base deletion resulting in uracil/uridine auxotrophy. By homology search using the *pabaA* gene from *A. nidulans* as template, a putative 4-amino-4-deoxychorismate synthase gene with locus reference number AO090701000057 was identified in the genome of *A. oryzae* RIB40 at AspGD [36]. A 950 bp upstream and 795 bp downstream flanking region was amplified by Phusion polymerase with oligonucleotides 1 and 2 for the up- and 3 and 4 for the downstream region using genomic DNA of strain RIB40 as template. The PCR fragments were cloned by in vitro recombination into a *Sma*I restricted pUC19 vector, which created a *Not*I restriction site between upstream and downstream flanking region. The resulting plasmid was linearised with *Not*I, dephosphorylated and gel purified. A URA-Blaster cassette was retrieved from plasmid *his*_SM-Xpress_URA [3, 6] by *Not*I restriction and ligated with the linearised pUC19 vector containing the *pabA* flanking regions. The resulting deletion cassette was excised from the pUC19 backbone by *Sma*I restriction and used for protoplast transformation of strain OP12 *pyrG*^−^ as previously described [6, 37]. Protoplasts were regenerated on media containing 1.2 M sorbitol and 7.3 µM *p*-aminobenzoic acid (paba). To test for paba auxotrophy, resulting transformants were replica-plated on media with and without the addition of paba. The homologous integration of the deletion cassette into the *pabA* locus in auxotrophic strains was further confirmed by PCR using oligonucleotides 5 and 6. To regain the uracil/uridine auxotrophy of the parental strain in Δ*pabA* strains, a marker recycling step was performed. Mitotic recombination within the URA-blaster cassette leads to the loss of the *pyrG* gene from the URA-blaster cassette and allows growth in the presence of 5ʹ-fluoroorotic acid (FOA). Solid minimal media were buffered with 20 mM HEPES to pH 7.0 and supplemented with *p*-aminobenzoic acid (7.3 µM), 10 mM uridine and 2 mg/ml FOA. Different amounts of conidia from selected transformants (range between 1 × 10^5^ and 1 × 10^6^ conidia) were plated and cultures incubated for up to 10 days at 28 °C. Conidia from colonies growing on the FOA-containing media were collected and checked for uracil/uridine and *p*-aminobenzoic acid auxotrophy. The resulting strains were named *A. oryzae* OP12_2Δ.

### Generation of SM-X_Strep-tag_URA and SM-X_Strep-tag_paba plasmids

To allow *N*- or *C*-terminal addition of a Strep-tag to recombinantly produced proteins, the *Not*I restricted plasmid *his*_SM-Xpress_URA [3, 6] was re-ligated to obtain a plasmid without selection marker, reducing the size of the plasmid by 3.5 kb. The overlapping oligonucleotides 7 and 8 encoding for the Strep-tag II core peptide WSHPQFEK [19, 38] were used to PCR-amplify the plasmid using Phusion polymerase. The PCR product was gel-purified and subjected to in vitro recombination to assemble the plasmid at the Strep-tag II sequence. The plasmid was then restricted by *Not*I to re-introduce the URA-Blaster cassette resulting in plasmid SM-X_Strep-tag_URA. In addition, the *pabA* gene was amplified with Phusion polymerase from genomic DNA of *A. nidulans* strain FGSC A4 using oligonucleotides 9 and 10. The resulting PCR-product was introduced into the *Not*I restricted Strep-tag II plasmid resulting in plasmid SM-X_Strep-tag_pabA. Restriction of either of the two SM-X_Strep-tag plasmids by *Nsi*I allows the generation of proteins with a *C*-terminal fusion of the Strep-tag II sequence, whereas restriction with *Nco*I allows the generation of *N*-terminally tagged fusion proteins.

### Heterologous expression of *acyN* in *A. oryzae* OP12_2Δ

A codon-optimised version of the *acyN* gene (*acyN*_OPT_) was synthesised by Eurogentec using the protein sequence from *A. sarcoides* strain NRRL 50072 (ID 6108) as template. For cloning into the SM-X_Strep-tag_URA expression plasmid, the gene was amplified by Phusion polymerase with oligonucleotides 11 and 12. In addition, the *acyN* gene was amplified from genomic DNA of strains NRRL 50072 and CBS 247.80 using primers 13 and 14. All PCR products were fused by in vitro recombination with the *Nco*I restricted SM-X_Strep-tag_URA plasmid. *E. coli* DH5α cells transformed with the expression plasmids were screened by colony PCR with Phire polymerase for the correct assembly using primers 15 and 16 for *acyN*_OPT_ and 17 and 16 for the genomic *acyN* versions. Plasmids were isolated and further checked by restriction digests. About 2.5 µg of each plasmid DNA was linearised with *Xba*I and used for protoplast transformation of the OP12_2Δ strain. Due to the constitutive expression from SM-X plasmids in OP12 strains, transformants showed different degrees of purple pigmentation and integration of the expression construct in these transformants was confirmed by PCR screening with the control primers described above.

### Co-expression of *acyN* and MO6277 in *A. oryzae* OP12_2Δ

The monooxygenase gene of MO6277 was amplified from genomic DNA of *A. sarcoides* CBS 247.80 with Phusion polymerase using oligonucleotides 18 and 19 and cloned by in vitro recombination into the *Nco*I restricted SM-X_Strep-tag_pabA plasmid. A *pyrG*^+^ and *pabA*^−^ *A. oryzae* OP12_2Δ strain harbouring the *acyN*_OPT_ expression construct and producing significant quantities of polyporic acid was used as receiver strain for the MO6277 gene. The MO6277_SM-X_Strep-tag_paba plasmid was linearised by *Xba*I restriction and used for protoplast transformation. Resulting transformants still produced a purple colour, but the colour appeared to diffuse into the medium rather than staying attached to the colonies. Integration of the expression construct in these transformants was confirmed by PCR using oligonucleotides 20 and 16.

### RNA extraction from *A. sarcoides* and semiquantitative RT-PCR analysis

Liquid PDB and ACM medium was inoculated with spores from *A. sarcoides* CBS247.80. In addition, PDB medium was also inoculated with spores from *A. sarcoides* wild isolate S1C. Cultures were incubated for 50 h under constant agitation at 150 rpm at 25 °C and a strong purple colour developed in the PDB cultures, whereas colouration was only at the early onset in ACM. Mycelium was harvested over Miracloth (Merk, Darmstadt, Germany), washed with water, pressed dry and shock-frozen in liquid nitrogen. The mycelium was ground to a fine powder and RNA was extracted using the MasterPure yeast RNA extraction kit as described in the manufacturer’s protocol. An additional DNAse treatment was performed and an aliquot of about 200 ng of the total RNA was subjected to PCR on the 3ʹ-region of the β-tubulin gene (ID 2686) with oligonucleotides 21 and 22 to ensure that all contaminating genomic DNA had been removed from the samples. About 3 µg of total RNA were used for first-strand cDNA synthesis using Superscript III reverse transcriptase and anchored oligo-dT primers. To adjust cDNA concentrations in all samples, PCR reactions were performed on the 3ʹ region of the β-tubulin gene by ensuring that the reactions did not run into saturation. The adjusted cDNA concentrations were then used for expression analyses of genes 6103 to 6109 with primers 23 to 36 (see Additional file 2: Table S1) to identify genes co-expressed with *acyN* (ID 6108). Subsequently, the cDNA was used to screen for the expression of all monooxygenase coding sequences with gene IDs: 523, 940, 1274, 3189, 3384, 3505, 3516, 4698, 5085, 6037, 6277, 7306, 7309, 7779, 8365, 8993, 9863 and 10,642. All primers 37–72 (see Additional file 2: Table S1) used for expression analyses were located at the 3ʹ-end of the respective gene and amplified a fragment of about 500 bp. Where possible, intron spanning sequences were selected. As primer efficiency and amplification control, genomic DNA of strain CBS 247.80 was used. PCR products were separated on a 1.5% agarose gel and an exposure time was selected that avoided pixel saturation of cDNA-derived PCR products using the QuantityOne software (Bio-Rad Laboratories).

### Enzyme purification

For purification of AcyN, *A. oryzae* strains producing AcyN with an *N*-terminal Strep-tag were grown for 27 h at 28 °C in 2 × 50 ml malt extract medium supplemented with 7.3 µM paba. Mycelium was harvested over Miracloth and pressed dry. Approximately 4 g of mycelium was ground to a fine powder under liquid nitrogen and suspended in 8 ml of buffer W (100 mM Tris/HCl pH 8.0, 150 mM NaCl, 1 mM EDTA). Cell debris was removed by 8 min centrifugation at 4 °C and 4000 rpm, followed by 2 min centrifugation of the supernatant at 4 °C and 13,000 rpm. The cell-free extract was subsequently filtered over a 0.45 µm filter (Sartorius) and applied to a Strep-Tactin Superflow gravity flow column (1 ml bed volume, IBA Lifesciences GmbH, Göttingen, Germany) previously equilibrated with buffer W. After washing the column with five column volumes of buffer W, AcyN was eluted with buffer W supplemented with 2.5 mM desthiobiotin. Eluates were concentrated using a centrifugal filter device (Amicon Ultra centrifugal filter units, Ultra-15, MWCO 30 kDa). Homogeneity of the purified enzyme was analysed by SDS-PAGE using a NuPAGE 4–12% Bis–Tris gel in a MOPS buffered running system (Invitrogen, Thermo Fisher Scientific).

### In vitro AcyN activity assays

In vitro reactions were performed as previously described for the aspulvinone E synthetase MelA [3] with some modifications: A 5 ml reaction contained 100 mM PIPES buffer pH 7.5, 6 mM ATP and 8 mM MgCl_2_, 0.2 mg of purified AcyN enzyme and either 2.5 mM *p*-hydroxyphenylpyruvate, 2.5 mM phenylpyruvate, or a combination of the two substrates (2.5 mM *p*-hydroxyphenylpyruvate and 2.5 mM phenylpyruvate, 2.5 mM *p*-hydroxyphenylpyruvate and 1 mM phenylpyruvate, or 1 mM *p*-hydroxyphenylpyruvate and 2.5 mM phenylpyruvate). Reactions without the addition of enzyme served as controls. After incubation at 28 °C for 24 h in the dark, reactions were acidified to pH 3 with HCl. This results in the precipitation of PIPES and proteins and the resulting pellet was separated from the supernatant by centrifugation. Both, pellet and supernatant fraction were extracted twice with ethyl acetate. Extracts were dried under reduced pressure, dissolved in methanol and analysed by HPLC as described above.

### Computational modelling

A model structure of monooxygenase MO6277 was produced with AlphaFold2 [22] via ColabFold—a Jupyter Notebook incorporated in Google Colaboratory [39]. The FASTA amino acid sequence of MO6277 was provided to the MMseqs2 server, which then aligns multiple sequences by searching the UniRef100 and environmental sequences databases with three profile-search iterations each. Finally, the five best models are ranked by AlphaFold2 applying a model confidence measure which indicates the prediction quality. Final protein models are relaxed using Amber force field (AMBER99SB-ILDN) [40]. An alternative structure prediction using I-TASSER (Iterative Threading ASSEmbly Refinement) was also applied [41] and used to identify the haem co-factor binding site. I-TASSER combines the multiple threading LOMETS (Local Meta-Threading Server) approach [42] against all available protein structures in the PDB with iterative template-based fragment assembly simulations to generate its protein model structures. It also applies COFACTOR [43] to annotate biological function and ligand-binding sites. Both structure prediction methods resulted in similar structures, with I-TASSER predicting a narrower binding pocket. Haem was subsequently superimposed into the best ranked AlphaFold structure for visualisation purpose. This AlphaFold structure was also used to predict tunnels towards the active site using CAVER Analyst 2.0 [44], which follows a Voronoi diagram-based analysis applying the standard tunnel computation settings with a minimum probe radius of 0.9 Å and the central haem binding cysteine residue as starting point.

## Supplementary Information

**Additional file 1: Figure S1. **HPLC analysis of supernatant extracts from AcyN in vitro reactions. This figure is supplementary to Fig. 5B in the main text. Numbers in brackets denote the concentration of substrates in (mM). PP = phenylpyruvate, *p*-OH-PP = *p*-hydroxphenylpyruvate. Cont = control reaction in the absence of AcyN. Positions of standards are indicated by grey bars. $ = *p*-hydrox-phenylpyruvate, # = phenylpyruvate. Trace amounts of atromentin are present in the supernatant fraction of the in vitro assay when *p*-hydroxphenylpyruvate is used as sole substrate.

**Additional file 2: Table S1.** Oligonucleotides used in this study.

## Data Availability

The sequence of the synthetic codon-optimised *acyN* gene is available under the GenBank Accession number OL770279. Homology model structures generated for the monoxyenase MO6277 can be retrieved freely available from the Figshare repository (https://doi.org/10.6084/m9.figshare.17186294.v1). *Ascocoryne sarcoides* strain NRRL 50072 is available upon request from the Agricultural Research Service Culture Collection (NRRL) and strain CBS 247.80 can be obtained from the CBS-KWAN collection at the Wsterdijk Fungal Biodiversity Institute.
