# Supplementary material for: Characterisation of ascocorynin biosynthesis in the purple jellydisc fungus Ascocoryne sarcoides
Source: Fungal Biol Biotechnol. 2022 Apr 27;9:8. doi: 10.1186/s40694-022-00138-7 (PMC9047271; doi:10.1186/s40694-022-00138-7)
Supplement: Supplementary file 1 — Additional file 1: Figure S1. HPLC analysis of supernatant extracts from AcyN in vitro reactions. This figure is supplementary to Fig. 5B in the main text. Numbers in brackets denote the concentration of substrates in (mM). PP = phenylpyruvate, p-OH-PP = p-hydroxphenylpyruvate. Cont = control reaction in the absence of AcyN. Positions of standards are indicated by grey bars. $ = p-hydrox-phenylpyruvate, # = phenylpyruvate. Trace amounts of atromentin are present in the supernatant fraction of the in vitro assay when p-hydroxphenylpyruvate is used as sole substrate. [file 40694_2022_138_MOESM1_ESM.pptx]

## Slide 1
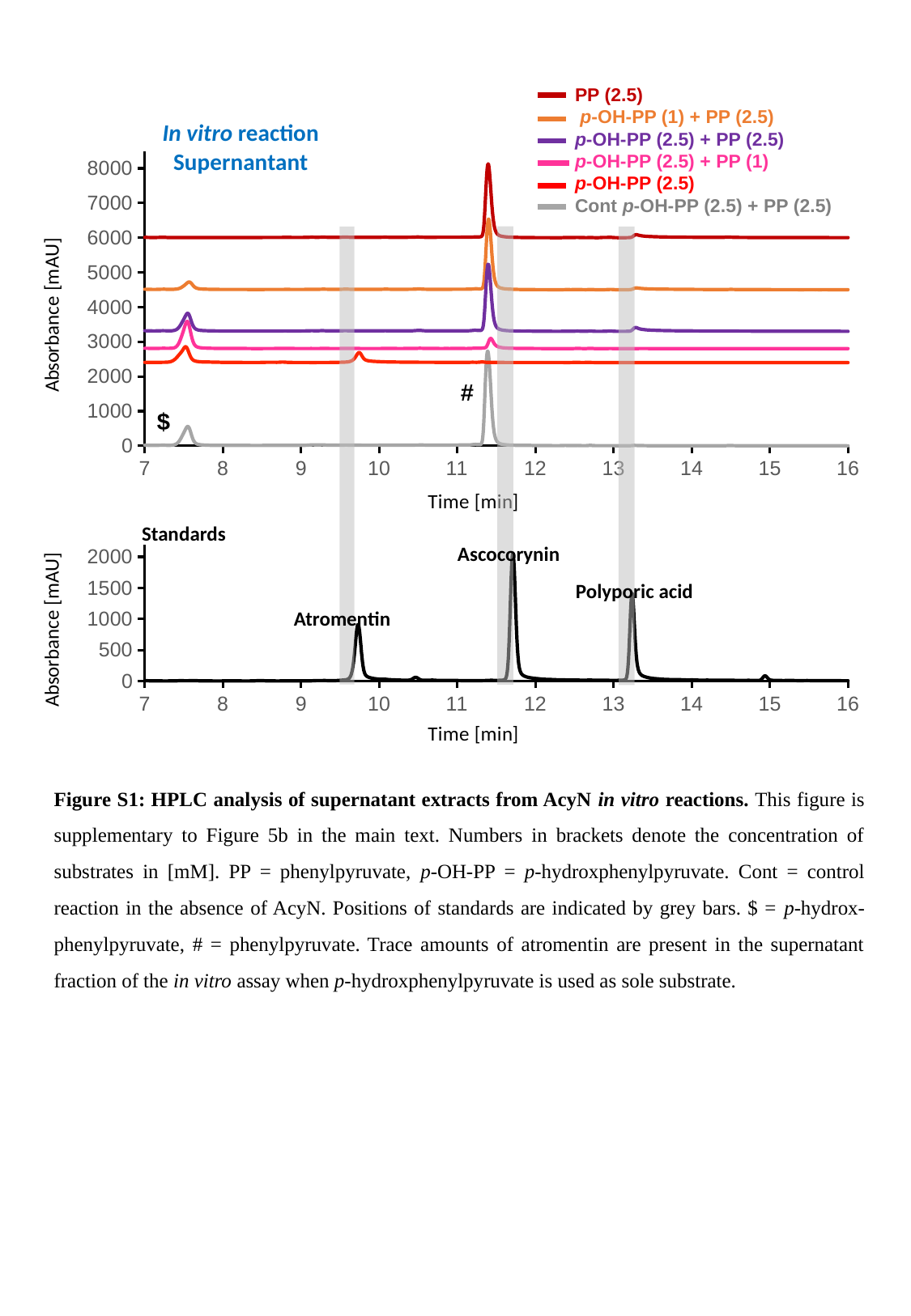

PP (2.5)
 p-OH-PP (1) + PP (2.5)
p-OH-PP (2.5) + PP (2.5)
p-OH-PP (2.5) + PP (1)
p-OH-PP (2.5)
Cont p-OH-PP (2.5) + PP (2.5)
In vitro reaction
Supernantant
### Chart
| Category | Supernatant Chromatogram Data: | Supernatant Chromatogram Data: | Supernatant Chromatogram Data: | Supernatant Chromatogram Data: | Supernatant Chromatogram Data: | Supernatant Chromatogram Data: |
|---|---|---|---|---|---|---|
#
$
Standards
Ascocorynin
### Chart
| Category | Clagr3.11 |
|---|---|Polyporic acid
Atromentin
Figure S1: HPLC analysis of supernatant extracts from AcyN in vitro reactions. This figure is supplementary to Figure 5b in the main text. Numbers in brackets denote the concentration of substrates in [mM]. PP = phenylpyruvate, p-OH-PP = p-hydroxphenylpyruvate. Cont = control reaction in the absence of AcyN. Positions of standards are indicated by grey bars. $ = p-hydrox-phenylpyruvate, # = phenylpyruvate. Trace amounts of atromentin are present in the supernatant fraction of the in vitro assay when p-hydroxphenylpyruvate is used as sole substrate.
