# Supplementary material for: Characterisation of ascocorynin biosynthesis in the purple jellydisc fungus Ascocoryne sarcoides
Source: Fungal Biol Biotechnol. 2022 Apr 27;9:8. doi: 10.1186/s40694-022-00138-7 (PMC9047271; doi:10.1186/s40694-022-00138-7)
Supplement: Supplementary file 2 — Additional file 2: Table S1. Oligonucleotides used in this study. [file 40694_2022_138_MOESM2_ESM.docx]

**Table S1:** Oligonucleotides used in this study

| **Number** | **Name** | **Sequence (5’ – 3’)** | **Purpose** |
| --- | --- | --- | --- |
| 1 | pabaAAory_up_f | ATTCGAGCTCGGTACCCGGGATA TAGAGTTTACCCCTACTG | Deletion *pabA* in *A. oryzae* |
| 2 | pabaAAory_up_r | ATGGTGCCAACAATCTGCGCCGT ATGGATGAACTGTAGC | Deletion *pabA* in *A. oryzae* |
| 3 | pabaAAory_down_f | GAGAAGCTCGACAACCTGAAAG CATGACCACATTCTTCCTC | Deletion *pabA* in *A. oryzae* |
| 4 | pabaAAory_down_r | CGACTCTAGAGGATCCCCGGGT CTCAACAAAATGG | Deletion *pabA* in *A. oryzae* |
| 5 | pabaAAory_upC_f | GTGCTCGAGTTTCGTCTCATG | Control primer for *pabA* deletion cassette |
| 6 | prpB_rv | CAGGTTGTCGAGCTTCTCTTC | Control primer for *pabA* deletion cassette |
| 7 | SMX-Streptag_f | CCCACCCCCAGTTCGAGAAGCC ATGGTAACAGCAGTGATTTC | Generation SM-X_Strep-tag plasmid |
| 8 | SMX-Streptag_r | TCGAACTGGGGGTGGGACCAAT GCATGGTGCTGTGATGAG | Generation SM-X_Strep-tag plasmid |
| 9 | PabaAAnIFSt-SMX_f | CTCGGTACCCGCGGCCGCGCTC TACGGCCTGACACC | Amplification *pabaA* from *A. nidulans* |
| 10 | PabaAAnIFSt-SMX_r | GAGGATCCCCGCGGCCGCGCTC TCCGCGACTATGAGG | Amplification *pabaA* from *A. nidulans* |
| 11 | AcyNOPT_IFSt-SMX_f | CAGTTCGAGAAGCCATGGATGGT CGCCCGCAACCTC | Amplification of *acyN*_OPT_ |
| 12 | AcyNOPT_IFSt-SMX_r | TCACTGCTGTTACCATGGTCAGA GGCCGCGTTCCTC | Amplification of *acyN*_OPT_ |
| 13 | acyN_Strep_f | GTTCGAGAAGCCATGGATGGTG GCCAGAAATCTCTAC | Amplification of *acyN* from *A. sarcoides* |
| 14 | acyN_Nco_r | ACTGCTGTTACCATGGTTACAAA CCCCGCTCTTCC | Amplification of *acyN* from *A. sarcoides* |
| 15 | AcyNopt_cont_f | CATCCAGACCGAACTCAAGC | Cloning control primer for *acyN*_OPT_ |
| 16 | Seq_AttrpC_rv | GAATTTTACCAGTGGCCTAGG | Cloning control primer in T*trpC* |
| 17 | acyN_cont_f | AGGATGAGGCAGCTAGATTG | Cloning control primer for *acyN* |
| 18 | AsMo6277If_f | CCAGTTCGAGAAGCCATGGATG CATTATTCTCCTCTCACAG | Amplification of MO6277 from *A. sarcoides* |
| 19 | AsMo6277If_r | AATCACTGCTGTTACCATGGTCA CTTAAAAATCACTCTGACGC | Amplification of MO6277 from *A. sarcoides* |
| 20 | AsMO6277_qPCR_f | GCTGGGAAAATTGAAGGAAGAG  T | Control and RT-PCR primer for MO6277 |
| 21 | As_bTub_cont_f | CAGATCTCCGAAAGTTGGCTG | RT-PCR primer for β-tubulin from *A. sarcoides* |
| 22 | As_bTub_cont_r | CATGTTGGACTCAGCTTCAGTG | RT-PCR primer for β-tubulin from *A. sarcoides* |
| 23 | AS6103_cDNA_f | CTGGCTTCTGGAGTGAACTTAC | RT-PCR 6103 |
| 24 | AS6103_cDNA_r | CTGTAGCGCCTCAGCTGTG | RT-PCR 6103 |
| 25 | AS6104_cDNA_f | GTACGCAGGCTATCAACTATATC | RT-PCR 6104 |
| 26 | AS6104_cDNA_r | CCTCCGCTTCGTCACTTCC | RT-PCR 6104 |
| 27 | AS6105_cDNA_f | CATCAAGGAAGATTGTGGTTATG | RT-PCR 6105 |
| 28 | AS6105_cDNA_r | GTGCACTTCCATTTCCAGCTG | RT-PCR 6105 |
| 29 | AS6106_cDNA_f | CGACGGAGAGTCAGGTGC | RT-PCR 6106 |
| 30 | AS6106_cDNA_r | CTCTTCTCACTGTAACTGCTTC | RT-PCR 6106 |
| 31 | AS6107_cDNA_f | CTCTCCTGGGCCAACATCAG | RT-PCR 6107 |
| 32 | AS6107_cDNA_r | GCTGCAGGTATCGACATGG | RT-PCR 6107 |
| 33 | AS6108_cDNA_f | CAGGCTACTCTTACGGCTC | RT-PCR 6108 |
| 34 | AS6108_cDNA_r | CAAACTCGCCCGTAGCGTC | RT-PCR 6108 |
| 35 | AS6109_cDNA_f | GACTTCATCTCTTACAATCACATC | RT-PCR 6109 |
| 36 | AS6109_cDNA_r | CGACAGCCAATTCGACGAC | RT-PCR 6109 |
| 37 | AsMO523cont_f | CCTACCATCTTCTGGATGCTC | RT-PCR MO523 |
| 38 | AsMO523StagIF_r | AATCACTGCTGTTACCATGGTC ATTCGGGTGCCATAGCC | RT-PCR MO523 |
| 39 | AsMO940cont_f | CGCCCAAACTCCTGTCATACT | RT-PCR MO940 |
| 40 | AsMO940StagIF_r | AATCACTGCTGTTACCATGGTCA TATCTTTTGACTTAGCCAAATAG | RT-PCR MO940 |
| 41 | AsMO1274_qPCR_f | ATCGAGAAGATGCGGGCTGA | RT-PCR MO1274 |
| 42 | AsMO1274_qPCR_r | CTAACCTAACACTCTCTTTGTGA | RT-PCR MO1274 |
| 43 | AsMO3189_qPCR_f | CAAAGGAGATCACGATGCAGA | RT-PCR MO3189 |
| 44 | AsMO3189_qPCR_r | TCAACAAGTTGGCGTCAACTTG | RT-PCR MO3189 |
| 45 | AsMO3384cont_f | CAAGATCGGTCCTTGGAGATT | RT-PCR MO3384 |
| 46 | AsMO3384ST_IF_r | AATCACTGCTGTTACCATGGTTA ACTCCGTCTCCTCCTCAA | RT-PCR MO3384 |
| 47 | AsMO3505_qPCR_f | GTCCCTATGCTGTTGATACGA | RT-PCR MO3505 |
| 48 | AsMO3505_qPCR_r | TTAGACAACTGATCCTACCGCA | RT-PCR MO3505 |
| 49 | AsMO3516_qPCR_f | GTGCTACAGAAGTTGCAGAGA | RT-PCR MO3516 |
| 50 | AsMO3516_qPCR_r | CAGCCCTCTTCCTAATTCCCA | RT-PCR MO3516 |
| 51 | AsMO4698_qPCR_f | CCTAGAGAAGCTCAAGAACCTA | RT-PCR MO4698 |
| 52 | AsMO4698_qPCR_r | GGCGCTTCAATCCAACCTTATA | RT-PCR MO4698 |
| 53 | AsMO5085_qPCR_f | GAGAGAATCGTCTACGAGACT | RT-PCR MO5085 |
| 54 | AsMO5085_qPCR_r | CCTTGGTGTGAAAATGCAAGAGA | RT-PCR MO5085 |
| 55 | AsMO6037_qPCR_f | GTTCTCAACAATCCACAGGTTCT | RT-PCR MO6037 |
| 56 | AsMO6037_qPCR_r | CTTCTTAAGGAGGACTCGGAGA | RT-PCR MO6037 |
| 57 | AsMO6277_qPCR_f | GCTGGGAAAATTGAAGGAAGAGT | RT-PCR MO6277 |
| 58 | AsMO6277_qPCR_r | GTTGCTCTGCATACTCGGCT | RT-PCR MO6277 |
| 59 | AsMO7306_qPCR_f | GCTCAGAGACTACTGCGAC | RT-PCR MO7306 |
| 60 | AsMO7306_qPCR_r | GTTTTCGCCAGAGAAGTTTCAAG | RT-PCR MO7306 |
| 61 | AsMO7309_qPCR_f | GCTCAAGCTTGAGCTCGAGT | RT-PCR MO7309 |
| 62 | AsMO7309_qPCR_r | CGAATCCTCACTCCCCTTGT | RT-PCR MO7309 |
| 63 | AsMO7779cont_f | CATTGCCTGGGAATCACTCGT | RT-PCR MO7779 |
| 64 | AsMO7779ST_IF_r | AATCACTGCTGTTACCATGGTTA CAGTATTTTCACACCAGTTCCA | RT-PCR MO7779 |
| 65 | AsMO8365_qPCR_f | CGAGGCGATGCGCCTTAAT | RT-PCR MO8365 |
| 66 | AsMO8365_qPCR_r | CCACCATTGGCCCATCCTTA | RT-PCR MO8365 |
| 67 | AsMO8993_qPCR_f | GATTCTCCACCCTGAAGTCCA | RT-PCR MO8993 |
| 68 | AsMO8993_qPCR_r | GGACATCTGCATACCTTGACA | RT-PCR MO8993 |
| 69 | AsMO9863_qPCR_f | CTCGACGCTTGCATTAAGGAA | RT-PCR MO9863 |
| 70 | AsMO9863_qPCR_r | CTATTGTAGCAGCTCCGGTTTA | RT-PCR MO9863 |
| 71 | AsMO10642qPCR_f | CCCGCATTCTCCCATGTTCAA | RT-PCR MO10642 |
| 72 | AsMO10642qPCR_r | CCAGCTTGCAAGTTTGATCCAA | RT-PCR MO10642 |
